# Digital tools and self-administered home blood tests: A convergent mixed methods pilot study

**DOI:** 10.1177/20552076251365063

**Published:** 2025-07-31

**Authors:** Lana Hebib, Emelie Gustafson Hedov, Maria Storgärds, Peder af Geijerstam, Marie Löf, Jason HY Wu, Lisa Kastbom, Karin Rådholm

**Affiliations:** 1 Department of Health, Medicine and Caring Sciences, 4566 Linköping University, Linköping, Sweden; 2 Department of Medical Sciences, 8097 Uppsala University, Uppsala, Sweden; 3 Symptoms Europe AB, Uppsala, Sweden; 4 214434 MinForskning AB, Uppsala, Sweden; 5 Department of Medicine, 27106 Karolinska Institute, Huddinge, Sweden; 6 The George Institute for Global Health, 7800 University of New South Wales, Sydney, Australia; 7 The School of Population Health, 7800 University of New South Wales, Sydney, Australia

**Keywords:** Decentralized clinical trials, digital health, mixed methods, digital clinical trials, remote patient monitoring, self-monitoring, mHealth

## Abstract

**Background:**

Digital tools and self-administered home blood tests offer a flexible approach to research data collection, biological sample management, and informed consent but require evaluation in relevant study settings. This study explored user experiences with a web-based digital tool for trial management, symptom reporting, and home blood testing.

**Methods:**

Forty-three middle-aged participants took part in a 12-week mixed-method study, using digital tools for trial management, self-reported health data, and self-administered glycated hemoglobin A1c home blood tests. Usability was assessed through the validated mHealth App Usability Questionnaire (MAUQ) with additional study-specific items. Every second participant completed a semi-structured interview, analyzed using qualitative content analysis.

**Results:**

The MAUQ responses (scale 1–7) indicated that digital consent (94.7% agreed), and home blood tests (100% agreed) were well-received. However, finding information on digital tools was challenging (52.9% disagreed it was easy), and participants did not perceive the tools highly effective for managing health (52.9% disagreed they were helpful). Interviews with 20 participants reinforced these findings, emphasizing motivation, support, efficient resource use, and the importance of clarity, usability, safety, and security.

**Conclusion:**

While self-administered blood tests and online consent were considered user-friendly, improvements are needed in digital tool navigation and information accessibility to enhance usability in decentralized trials, especially for participants who find them difficult or unhelpful.

## Introduction

Decentralized clinical trials, which facilitate research without traditional clinical trial sites and physical contact between research teams and participants in some or all trial activities, are supported by digital technology. These trials offer many potential benefits to patients, such as reduced burden, and contribute to the efficiency of conducting clinical trials, leading to the European Medicines Agency (EMA) and the United States Food and Drug Administration providing regulatory guidance to further facilitate the conduct of decentralized clinical trials.^1,2^

The emergence of digital tools is one key reason that has supported the use of decentralized trials. Such tools provide benefits, including convenience and easier access to information and to health data as well as facilitating clinical trial participation.^2–4^ Similarly to digital tools, usage of a validated self-administered home blood tests in decentralized clinical trials may reduce the burden on participants (avoid having to attend pathology centers or study sites), requiring less resources as well as providing minimal invasiveness, low cost, easy storage, and handling.^5^

Despite the recognition that digital tools and self-administered home blood tests offer many potential benefits, their design and deployment are often done without the participation of the intended users^4,6^ and may thus not meet their needs.^7^

Studies of users’ and patients’ perspectives on digital tools are few,^8^ and prior literature indicates there may be a low willingness among patients to use such tools for healthcare purposes.^9^ Therefore, qualitative research may add information on what users want and find helpful and facilitate the development of tools that meet their needs.^10^ Digital health tools may be evaluated through usability questionnaires.^11^ The mHealth App Usability Questionnaire (MAUQ) is a validated questionnaire, specifically designed to evaluate the usability of mobile health (mHealth) tools.^11^

The digital tools *minforskning.se* (MinForskning AB Uppsala, Sweden), for research trial participant management, and *Symptoms* (Symptoms Europe AB, Uppsala, Sweden), for patient-self reporting and digital health data,^12^ will be used in an upcoming randomized controlled clinical trial using mHealth tools to support beneficial changes in dietary habits of individuals with type 2 diabetes (T2DM). These platforms are relatively new and developed for usage in clinical research, but the users’ perspectives on the platforms have not been evaluated in previous studies. Therefore, the aim of the study was to, in a Swedish context, investigate the participants’ experiences of the digital tools, *minforskning.se* and *Symptoms*, and self-administered home blood tests in preparation of the DIgitAl diabetes Treatment–the Healthy Eating, heaLthy Patients (DIATEST-HELP) trial by use of a convergent mixed methods study, to identify potential facilitators and barriers that may arise with the use of these digital tools.

## Materials and methods

### Participants

**Figure 1.**
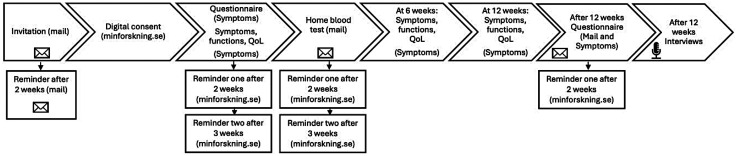
Steps of the convergent mixed methods study in preparation of the DIgitAl diabetes Treatment—the Healthy Eating, heaLthy Patients (DIATEST-HELP) trial.

**Figure 2.**
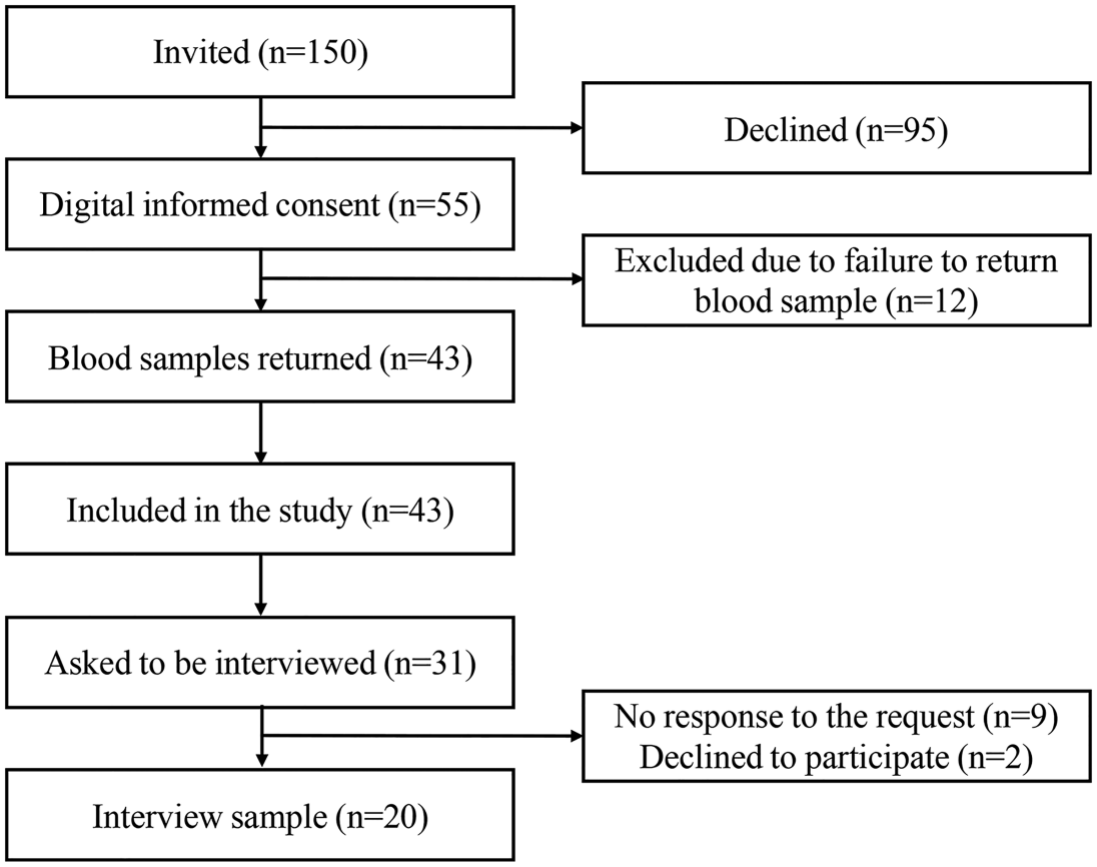
Flowchart of the study.

For this pilot study, aiming to explore feasibility, usability, and participant experiences, invitation letters were sent to 150 randomly selected participants from the general population who took part in the Uppsala The Swedish CArdioPulmonary bioImage Study cohort and had accepted to be asked to participate in other studies.^13^ In addition, inclusion criteria were access to a smartphone, tablet, or computer. Exclusion criteria were not having access to *BankID* (Finansiell ID-Teknik BID AB, Stockholm, Sweden), a digital identification system used by 92% of the population in Sweden,^14^ not being able to write or read Swedish, and/or permanent residency outside of Sweden. Participants were required to return a blood sample to participate in the qualitative interview study (Figures 1 and 2). The study was approved by the Swedish Ethical Review Authority (Dnr.: 2022-03797-01).

### The web-based clinical trial *minforskning.se*

Participants received digital invitations containing a uniform resource locator and a quick response (QR) code to the web-based clinical trial platform *minforskning.se* containing information about this pilot study and an option to give written informed consent (Supplemental figure 1). The website contained contact information to digital support via e-mail. Phone calls were made if needed to support participants when navigating the digital tools. The number of questions asked was monitored. The system guided participants to the digital tool *Symptoms*. The digital tool for trial participant management (*minforskning.se*) provided participants with test results from the self-administered home blood tests used in the study. This was done securely through a non-integrity-restricted e-mail sent from *minforskning.se*, which only contained information prompting the participant to log in. The results were only accessible after the participants’ login using *BankID*.

### The digital health data collection platform *Symptoms*

Participants were asked to complete a questionnaire about demographics, household characteristics, lifestyle, medical history, and medications on *Symptoms*, a web-based health data collection platform for patient-reported symptoms, functions, and quality of life (QoL) to be used on smartphones, tablets, or computers, within 5 days of digital consent (Supplemental figure 2). *Symptoms* allowed participants to report symptoms, functions, and QoL, as often as they liked for 12 weeks and when prompted to do so, at 6 and 12 weeks. Reminders were also sent after 2 and 3 weeks. The system also allowed participants to draw symptoms on three-dimensional manikins and write free-text notes.^12^

### Self-administered home blood tests

All participants received a validated self-administered home blood test sample kit for collecting dried blood spots which contained instructions, two lancets, filter papers, and a return envelope. The participants were instructed to collect two to three droplets of capillary blood on each of the filter papers and return these by ordinary mail within 2 weeks of survey completion on *Symptoms*. The dried blood spots could be kept at room temperature for 2 weeks, which enabled the use of regular post for transport. There were no personal data in the self-administered home blood test sample kit. The kit only contained a code, to which the key was accessible exclusively by the study coordinator.

Filter papers with dried blood spots were analyzed for glycated hemoglobin A1c (HbA1c) at time of biospecimen arrival at *Vitas Analytical Services* (Oslo, Norway). *Uppsala BioLab* (Uppsala, Sweden) handled the samples. The results of the HbA1c analyses were available to the study participants at *minforskning.se* at the end of the quantitative data collection (at 12 weeks).

### The MAUQ

After 12-week participation, study participants received the validated 21-item questionnaire MAUQ,^11^ with an option to provide free-text comments, to assess usability of the digital tools (Supplemental methods). Additional questions on the usability of self-administered home blood testing and the digital tools tested were added to the questionnaire. The additional questions were validated using brief cognitive “think aloud” interviews with five individuals to make sure that the additional questions were easy to understand and respond to, from a respondent perspective.^15^

The questionnaire was sent to participants on paper via mail. Participants also received an e-mail, prompting participants to log in to *minforskning.se* which contained a link to *Symptoms*, where they could access the questionnaire digitally.

### Semi-structured individual interviews

The sampling of the qualitative interview study was purposive. Every second person of the total study population was asked to participate. We aimed to recruit approximately 20 participants to reach data saturation, or redundancy, i.e. conducting additional interviews is not considered to add new views and perceptions.^16^ After conducting 20 interviews, saturation/redundancy was deemed to have been reached. This assessment was made through discussions between the interviewer (LH; female, physician, general practitioner (GP), PhD student) and the supervising senior researcher (LK: female, physician, GP, PhD), who has extensive experience in qualitative methodology. Verbal informed consent was recorded from each participant before the interview started.

An interview guide (Supplemental material) with open-ended questions regarding smartphone usage, support in chronic illnesses, self-administered home blood tests, the digital tools used in the study (*minforskning.se* and *Symptoms*), and digital tools in general was created by the research group.^16^ The guide was tested in the first interview (pilot interview), which was subsequently included in the current study, as it was considered to be of good quality. Clarifying questions^17^ were asked based on answers to assure understanding and minimize the risk of misunderstanding.

The individual interviews were conducted between 28 November 2023 and 22 January 2024, after the quantitative data collection was finished. LH conducted all the interviews by telephone. The interviewer had no prior relationship with the participants. The interviews were digitally recorded and transcribed verbatim by *Space360* (Norsborg, Sweden). Interviews varied in length between 15 and 40 min.

## Analysis

Continuous variables were presented as means with standard deviations and categorical variables were presented as frequencies with percentages. Differences regarding age and gender between the responders and non-responders of MAUQ were analyzed using Chi-square test and Wilcoxon rank sum test.

### The MAUQ

The MAUQ was presented in its entirety to the participants, but one item was irrelevant to the study and thus excluded in the analysis (Q14- “The app improved my access to health care services”). Each of the remaining items in the questionnaire, as well as five additional study specific, was assigned a score ranging from 1 (disagree entirely) to 7 (agree entirely) and a value of 4 indicating neither agreement nor disagreement. Data was presented as percentages of agreement (>4), neither agreement nor disagreement (=4) and disagreement (<4).

### Semi-structured individual interviews

**Figure 3.**
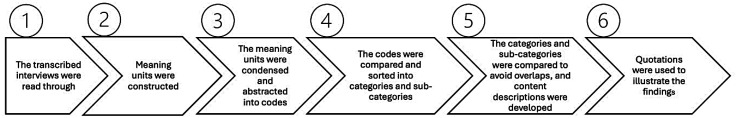
Steps of the qualitative content analysis.

The transcribed interviews were analyzed through manifest inductive qualitative content analysis.^18^ The six main steps of the analysis are illustrated in Figure 3. Examples of the analysis process are shown in Table 1. The analysis was carried out individually by the first (LH) and shared last author (LK), with extensive experience in qualitative research. They compared separately created codes and categories, which were later discussed and revised by the first and last authors (LH, LK, and KR; female, physician, GP, PhD, associate professor).

**Table 1.** Examples of the steps of the analysis using qualitative content analysis.

Meaning unit	Code	Sub-category	Category
Wanting to be able to ask questions regarding your health	Health questions	Self-interest is crucial	What's in it for me
Healthcare does not become so loaded, they have enough	Put load off healthcare	Limited resources in the healthcare	Efficient use of recourses
Someone to call if there has been a hassle	Telephone support	Alternatives to digital solutions and social interactions are requested	The support of another person is irreplaceable

## Results

### Characteristics of the participants

Of the 150 individuals that were invited, 55 consented to participate and 43 of these (78%) returned the blood samples and were included in the study (Figure 1). The mean age was 64 years, 67.4% were women (Table 2). All 43 participants had normal HbA1c values, i.e., below 42 mmol/mol (Table 2). For the individual qualitative interviews, some study participants did not respond to the interview request (n = 9), while others declined to participate due to lack of time (n = 2). Of the 20 participants who were interviewed, 65% were women, and most lived in urban areas (80%) and had a university education (70%) (Table 2).

**Table 2.** Demographics and characteristics of the participants.

	Participants (n = 43)
Women	29 (67.4)
Age, years, mean (SD)	64 (3.9)
Highest completed level of education -Did not complete primary school -Primary school -High school -College or university	5 (55.6) 2 (22.2) 2 (22.2) 0
Glycated hemoglobin, mmol/mol	36.8 (3.1)

	Interviewed participants (n = 20)
Women, n (%)	13 (65)
Age, years, mean (SD)	65 (4.2)
Living in urban area, n (%)	16 (80)
Cohabitant, n (%)	16 (80)
Having children, n (%)	18 (90)
University education, n (%)	14 (70)

Categorical variables are in numbers and percentages and continuous variables in mean and standard deviations. The missing rate was none for all variables except highest completed level of education, for which the missing rate was 36 (80%).

### Task completion and digital support

Table 3 shows the completion rates for the questionnaires on the digital platforms as well as the self-administered home blood tests. Of 55 blood sample kits, 43 (78%) were returned for analysis. All the returned blood tests were sampled correctly and used to analyze HbA1c. Of these, 24 (56%) were returned without a reminder, 19 (44%) were returned after reminders, but 12 (28%) participants did not return the self-administered home blood tests at all even though they had received three reminders during a period of 6 weeks. For the digital questionnaire about QoL, the median number of responses per participant was 2 (1–5). For the MAUQ, 17 (40%) participants responded digitally. All participants received MAUQ on paper as well, and 4 (9%) of these were returned. One individual responded on paper while another responded both digitally and on paper. Two of the responses on paper could not be identified, and these participants may therefore have responded both digitally and on paper. Digital support received 52 e-mails in total, of which 6 (12%) concerned difficulties logging on to *minforskning.se*, 6 (12%) concerned *Symptoms*, 3 (6%) were requests for test results available on *minforskning.se*, and 11 (21%) were general questions about the study.

**Table 3.** Task completion rates and digital support.

Task completion	n (%)
Informed consent	55 (37)
Blood samples returned to lab and total number of participants included in the study	43 (78)
Blood samples returned without reminder	24 (56)
Blood samples returned after reminder 1 (at 7 days)	6 (14)
Blood samples returned after reminder 2 (at 14 days)	7 (16)
Blood samples returned after reminder 3 (at 21 days)	6 (14)
Blood samples not returned after third reminder	12 (28)
MAUQ questionnaire on paper returned	4 (9)
MAUQ digital questionnaire returned	17 (40)
Digital baseline questionnaire	39 (91)
Digital questionnaire about quality of life (QoL) returned (39 invitations sent at 6 and 12 weeks)	
After 6 weeks:	19 (49)
-Returned within 7 days before first reminder	18 (46)
-Returned after second reminder at 21 days	1 (3)
After 12 weeks:	24 (62)
-Returned within 7 days before first reminder	21 (54)
-Returned after second reminder at 21 days	3 (8)
E-mail correspondence to digital support	N (%)
Total number of received e-mails by digital support	52 (100)
E-mails regarding difficulties login to *minforskning.se*	6 (12)
E-mails regarding difficulties login to *Symptoms*	3 (6)
Requests for test results	4 (8)
General questions about the study	11 (21)

The denominator for task completion was all invited individuals (n = 150), and for e-mail correspondence, the denominator was the total number of received e-mails (n = 52).

### MAUQ

Of all included participants, 21 (38.1%) returned the MAUQ. There were no significant differences in gender and age between the responders and non-responders (*p* = 0.376 and *p* = 0.151). Tables 4 and 5 show the results from the MAUQ and study-specific additional items. Most of the participants were comfortable consenting to participate digitally (94.7% agreed, 5.3% disagreed) and joining the study (84.2% agreed, 15.8% neither agreed nor disagreed) via *minforskning.se.* Furthermore, the instruction for self-administered blood home tests was found to be clear and easy to understand (100% agreed). The participants were comfortable to prick their finger for the self-administered blood test (89.5% agreed, 10.5% disagreed) and could draw enough capillary blood for the blood tests (89.5% agreed, 10.5% disagreed). Meanwhile, the average user did not find the information on the digital platforms well organized, so that the information would be easily found (35.3% agreed, 11.8% neither agreed nor disagreed, 52.9% disagreed it was easy). Furthermore, the digital tools were not seen as helpful tools to manage health efficiently (23.5% agreed, 23.5% neither agreed nor disagreed, 52.9% disagreed they were helpful).

**Table 4.** Responses of the mHealth App Usability Questionnaire (MAUQ).

Item	Agree, n (%)	Neither agree nor disagree, n (%)	Disagree, n (%)
Q1. The app was easy to use	7 (41.2)	2 (11.8)	8 (47.1)
Q2. It was easy for me to learn to use the app	7 (41.2)	2 (11.8)	8 (47.1)
Q3. The navigation was consistent when moving between screens *(n* *=* *16)*	7 (43.8)	2 (12.5)	7 (43.8)
Q4. The interface of the app allowed me to use all the functions (such as entering information, responding to reminders, viewing information) offered by the app	7 (41.2)	4 (23.5)	6 (35.3)
Q5. Whenever I made a mistake using the app, I could recover easily and quickly *(n* *=* *16)*	7 (43.8)	6 (37.5)	3 (18.8)
Q6. I like the interface of the app *(n* *=* *16)*	9 (56.3)	4 (25.0)	3 (18.8)
Q7. The information in the app was well organized, so I could easily find the information I needed	6 (35.3)	2 (11.8)	9 (52.9)
Q8. The app adequately acknowledged and provided information to let me know the progress of my action	7 (41.2)	2 (11.8)	8 (47.1)
Q9. I feel comfortable using this app in social settings	7 (41.2)	5 (29.4)	5 (29.4)
Q10. The amount of time involved in using this app has been fitting for me	10 (58.8)	4 (23.5)	3 (17.6)
Q11. I would use this app again	9 (52.9)	1 (5.9)	7 (41.2)
Q12. Overall, I am satisfied with this app	7 (41.2)	4 (23.5)	6 (35.3)
Q13. The app would be useful for my health and well-being	8 (47.1)	4 (23.5)	5 (29.4)
Q15. The app helped me manage my health effectively	4 (23.5)	4 (23.5)	9 (52.9)
Q16. This app has all the functions and capabilities I expected it to have *(n* *=* *16)*	3 (18.8)	9 (56.3)	4 (25.0)
Q17. I could use the app even when the Internet connection was poor or not available *(n* *=* *16)*	2 (12.5)	12 (75.0)	2 (12.5)
Q18. This mHealth^a^ app provided an acceptable way to receive health care services, such as accessing educational materials, tracking my own activities, and performing self-assessment.	6 (35.3)	8 (47.1)	3 (17.6)

a mHealth: mobile health.

Participants responses were divided according to: agree, > 4; neither agree nor disagree =4: disagree, < 4. Number of respondents = 17.

**Table 5.** Responses of the study-specific items added to the mHealth App Usability Questionnaire (MAUQ).

Item	Agree, n (%)	Neither agree nor disagree, n (%)	Disagree, n (%)
Q19. I feel comfortable consenting to participate in research studies digitally via *minforskning.se*	18 (94.7)	0 (0.0)	1 (5.3)
Q20. It was easy to join the study and get reminders via *minforskning.se*	16 (84.2)	3 (15.8)	0 (0.0)
Q21. I feel comfortable to prick myself in a finger at home (for the blood sample)	17 (89.5)	0 (0.0)	2 (10.5)
Q22. The instructions for the home blood tests were clear and easy to understand	19 (100.0)	0 (0.0)	0 (0.0)
Q23. It went well getting enough blood to fill the circles for the home blood tests	17 (89.5)	0 (0.0)	2 (10.5)

Participants responses were divided according to the following: agree, >4; neither agree nor disagree, =4; disagree, <4. Number of respondents = 19.

### Interviews

**Figure 4.**
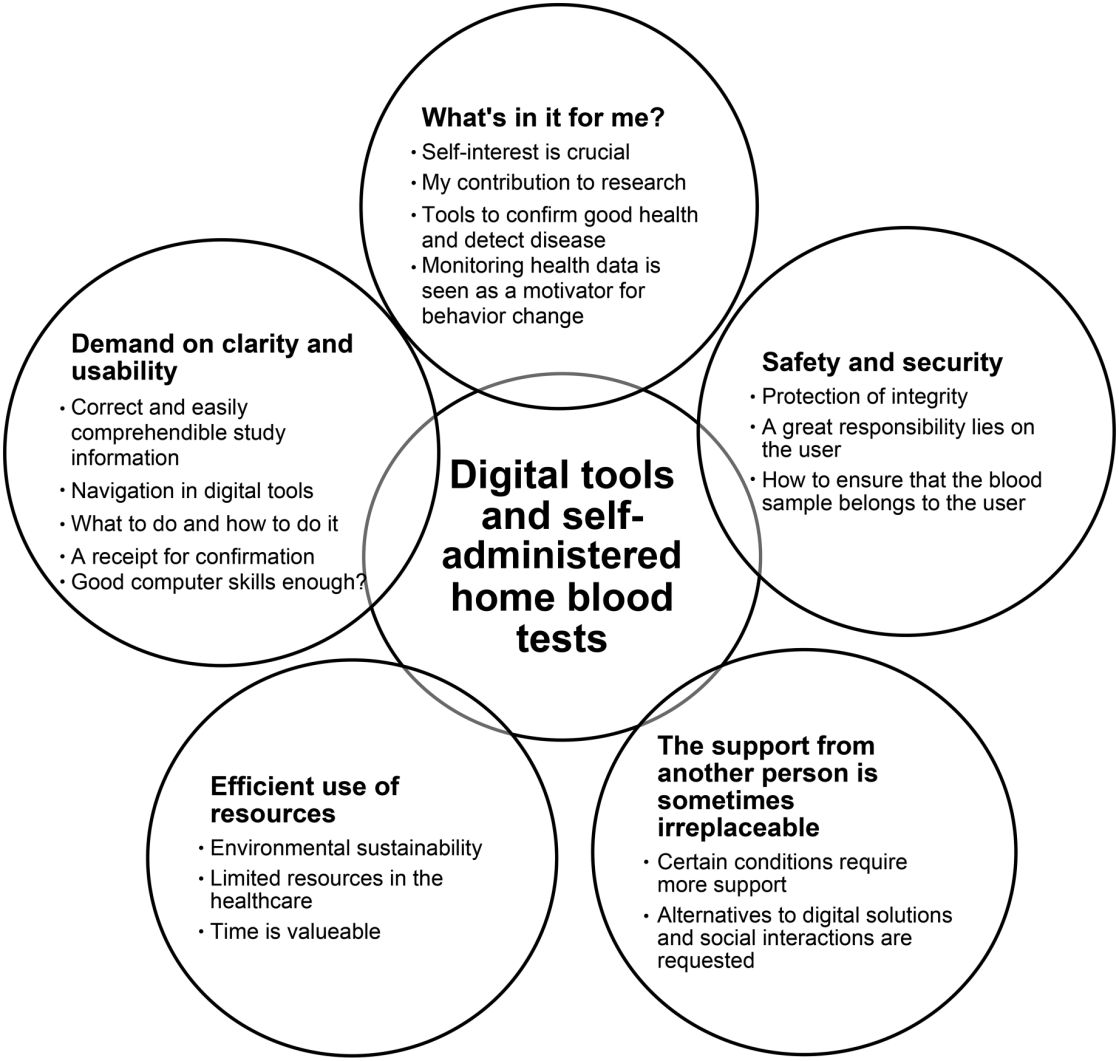
The 5 categories and 17 sub-categories illustrating the participants’ experiences of digital tools and self-administered home blood tests.

The following five categories were identified when analyzing data using manifest qualitative content analysis^18^; *what's in it for me?*, *safety and security*, *the support from another person is sometimes irreplaceable*, *efficient use of resources*, and *demand on clarity and usability* (Figure 4).

#### What's in it for me?

The category consists of four sub-categories describing the participants’ views on the importance of the user's motivation and needs.

##### Self-interest is crucial

Participants stressed the importance of their own needs and benefits when using digital tools as well as self-administered home blood tests. When the participants found the tool useful or if the tool provided benefits to them, they were more likely to use it. When the user was able to ask questions and gain knowledge of a certain condition that they themselves, or someone they knew, was affected by, this was seen as a major benefit. Understanding the purpose of the tool was also crucial. It must make sense to the user, according to the participants, who viewed that this was not the case when reporting symptoms. It was unclear what the information was being used and needed for, which created confusion and made participants less likely to complete the questionnaires:

“What's in it for me?” What do I gain from turning this in? Do I find out the results of this I have turned in then you are more likely to…if you get to know a little the results of the research. Maybe on an individual level but also on a group level, like what does it bring to contribute with this. (Participant 18)

I have never experienced any problems with that and not had any symptoms or anything like that. So that I…yes, then you can get the feeling that oh, am I the right person to answer these questions? (Participant 9)

The lack of symptoms made it difficult to see the point of using *Symptoms* and fill out questionnaires. The interpretation of the word *symptom* was described by one participant:

Like I was healthy so I did not have any symptoms so there no and I, I, have headaches really often och partly I forgot och had I not forgotten but I had probably thought I cannot go in there each and every other day and write that I have headaches like. But I should have done it of course. Because I had like learnt this and. And then I think symptom is a little strange word to, to me symptom is something then you have a disease och then for an example if you have a runny nose because you have the disease a cold and are you not ill then you do not like have any symptoms but then yes then you have discomforts or something. (Participant 1)

Many did not understand the purpose of the digital tools used in the study and when things did not make sense, they were unwilling to execute what was being asked of them in terms of questionnaires:

The purpose is surely important. What is it that I…? And clearness and that you need…If it is a tool that is not a survey but…You want to feel that you yourself have benefit from it in that case and it should be easy and easily understood too. (Participant 15)

The purpose of the use of mobile phones is to benefit the user and the urgency and importance decides when the phone is used, and notifications are limited, according to the participants:

But otherwise I try to keep away this with notifications and like that, because I know that it is not got that you get interrupted all the time, or that you lose your attention and like that…And when I am at work, then I put away my private phone pretty much. I have it with me in case someone in the family would call and it would be something urgent, but otherwise I do not sit and look at the private and notifications there. And on the work phone I do not have notifications on at all. (Participant 20)

##### My contribution to research

Participation in studies was viewed as a contribution to research and to those who benefit from it. This contribution was motivating for some and could in some cases lead to usage of tools that did not necessarily provide benefits directly to the user him- or herself:

It is surely good if you can do research so that there will be better medicines and increase quality of life for people. And you can detect diseases quicker, I can imagine. (Participant 4)

##### Tools to confirm good health and detect disease

Using tools to detect diseases was seen as a motivator. Many chose to participate to gain knowledge about their own health in general and blood glucose levels in particular. They also described usage of digital tools to monitor blood pressure. Receiving information about test results was important, and not being able to gain access to the results was a source of frustration for many as test results were difficult to find on *minforskning.se*. There was also a sense of urgency to receive test results. Participants asked for reassurance that the levels were normal as soon as possible:

Yes, it was that it took a very long time before I got the results. Now, I am not at all worried of the result so it did not really matter but one can imagine if you had high blood sugar or type 2 diabetes or like that, then you want the results, like, as soon as possible. (Participant 1)

I think it is great if you can get help with screening and detect health issues in time. (Participant 20)

##### Monitoring health data is seen as a motivator for behavior change

Participants had experience in using apps monitoring physical exercise, such as step trackers, and found it motivating. Digital tools monitoring pulse, sleep, and blood pressure were also commonly used. Many described that those tools contributed to a healthy lifestyle. Given the opportunity to monitor health data was also motivating when choosing to participate in studies:

The only thing I have used is a step tracker, one of those health apps. And then it has been connected to physical activity and step tracker and how much I exercise, I think it was also…it has been a while now, but then is was just I think I that registered myself, if I had exercised and what I had exercised….You get a little positively charged and then you exercise another time that week. (Participant 11)

However, many perceived that there is a limit and that information and monitoring could be too excessive. Some worried too frequent monitoring led to anxiety and even less awareness of the body and being less prone to noticing changes themselves, while others thought it may contribute to an addiction to the phone:

I have one of those with steps…that measures steps. That I think is…that is probably a lot to motivate me to move. That can help me to feel a little good then, if I have walked a lot of steps (laugh). //I do not want to have one of those pulse watches and everything like that, sleep measurers and like that//If it is only about health stuff, then I need to keep track of my own body first and foremost. It feels…To me it would…I almost think that there is something a little unhealthy when it gets a little too much of that, to measure the pulse and keep track in that way. For my part I think so. Yes, I mean that you might look more at that app then then, that “now I have slept this bad tonight” or so instead of to…”Am I tired or not?” I can feel that myself? Yes, and that it becomes an increasing phone addiction I think as well. (Participant 15)

#### Safety and security

The category consists of three sub-categories with emphasis on safety and security.

##### Protection of integrity

Concerns were raised about ensuring that the personal information was in the right hands and used for the right purposes. Many expressed that they wanted clear information about what their data was being used for. Participants were well aware of fraud online. For the same reason, the use of a mobile identification system was preferred for its convenience, rather than passwords and usernames, since the participants often found it difficult to keep track of different passwords. Some raised concerns about being able to rely on regular mail for sending blood samples and mentioned they might get lost in transportation:

just that it is safe and goes well. I can not see any disadvantage…The mail delivery maybe…Or mix-ups or…Yes, I do not know. Everything is possible when people are involved. (Participant 12)

Everything must be safe, particularly when it comes to illnesses and integrity and these things. You have to be protected in some way, so that it does not leak to anyone or it might get in to the wrong hands or whatever. I do not know what could happen, but times are like that now. (Participant 17)

##### A great responsibility lies on the user

Uncertainty, regarding the handling of the blood sample and whether the results would be affected by any inaccurate handling of the sample, was raised. Some expressed concern regarding contamination. Others had difficulties getting enough blood for the sample. Two kits for blood sampling were appreciated by some, in case there were issues. Participants appreciated that there were few steps in the process:

I don’t really know if you can contaminate. It depends how sensitive it is of course, the samples, how you handle them? (Participant 11)

##### How to ensure that the blood sample belongs to the user

Participants mentioned it might be difficult to ensure that the blood sample belongs to the user. However, none of the participants could see why the user would have a reason to ask someone else for a sample in this setting. Furthermore, participants expressed that important information about the participant may be missed out without a physical meeting:

The only thing could be that you do not have a real check on this person that…it could be someone else in the household. It is something like that then, or…that could…but some cheating then if you put it like that. But I do not really know, why, you would cheat there. (Participant 9)

#### The support from another person is sometimes irreplaceable

Two sub-categories exemplify the limitations of digital solutions.

##### Certain conditions require more support

It was clear that chronic illnesses, older people, and people with special needs were perceived to be unlikely and, in some cases, even uncapable of handling the digital tools and self-administered home tests used in the present study. Participants highlighted the need for additional support customized to the individual needs of the user. Particularly older people were often believed to be less-experienced users of digital tools. Similar thoughts as with digital solutions were expressed regarding self-administered home blood tests. While participants often found it simple to collect blood samples, they imagined that some may need additional support. Fear of needles was seen as an obstacle, and some had been assisted by another person. Using a needle on oneself was described as contra intuitive. Some had difficulties getting the required amount of blood. The skills required to handle needles were also mentioned, and the option of having a demonstration, or even assistance by healthcare staff was requested. However, the preference of having the option of a physical meeting with another person was also due to the wish to have a personal connection and to be seen by someone:

…but yes, to the extent it is a complement to the human contact I think it can be great…I think it should be a complement to the human if you say it like that, but then that you not…yes, feel that you are left alone if you say it like that …I think elderly that…yes, but there I think you need to have a…yes, a phone voice if you say it like that. Absolutely. (Participant 9)

If you are even older than me and maybe even more unused to these smartphones and that, then it is a big difficulty today to be older, and you almost get a little disabled if you cannot handle all these systems. It is a difficulty, that we discriminate those that are older and maybe have disabilities that cannot really handle this…I think that it maybe is not possible for them to do these at home blood tests then, if you are not used to computers or used to these systems, if I cannot even do it. (Participant 11)

##### Alternatives to digital solutions and social interactions are requested

While digital solutions were seen as easily accessible tools, they were seldom viewed to be enough as the only option available. The participants saw digital tools to detect disease and to get in contact with healthcare, and not as an alternative that stands on its own or is enough to deal with complicated conditions. The need for physical assistance by another person or through a phone call was seen as a necessity. Having someone to ask was non-negotiable. Although participants were aware of the digitalization in society, they were also concerned about those who might be excluded if they did not have access to such tools.

You can…Sure, you can do things on the computer and those things, and I do, but some things you want to address personally. Someone that listens to you, someone that sees how you are feeling when you say the things, and someone that can say “how does it feel now?” Because otherwise then…It becomes…On the computer it becomes a little unpersonal. (Participant 4)

#### Efficient use of resources

The category consists of three sub-categories highlighting resources.

##### Environmental sustainability

Participants described awareness of the environment and found the extra materials used to be wasteful. Many did not see the need for two packages for self-administered home blood tests, although others preferred having two kits in case there was a need. Furthermore, the plastic packaging was questioned. Advantages were seen with home blood sampling as it did not require any traveling and therefore protected the environment:

You sent out more than needed and I am a little…don’t think you should use nature's resources if not necessary, so maybe it felt a little unnecessary to send two of everything. (Participant 3)

##### Limited resources in the healthcare

Some were aware of the strains on healthcare and its limited resources and saw digital solutions and self-administered home blood tests as a way of protecting those limited resources. Lower costs for not using healthcare staff for blood sampling were mentioned:

There are lower costs, because it is much cheaper if you do this work yourself than if a nurse is doing it. (Participant 3)

##### Time is valuable

Self-administered home blood tests were seen as convenient, and the participants particularly appreciated the use of ordinary mail for transportation. Flexibility and having the option to decide when to take the test, not having to schedule appointments in healthcare, travel to healthcare facilities, and wait for the appointment, were seen as major benefits:

if you for an example have difficulties going to a healthcare centre, you live out on the countryside or whatever it now might be, then we have another bigger advantage of course, then when you live like me, I am pretty close to a healthcare centre, so that also contributes. (Participant 6)

#### Demand on clarity and usability

Five sub-categories that emphasize the importance of clarity and usability were identified.

##### Correct and easily comprehendible study information

The participants were not clear on the study's purpose and what was expected of them. They requested information to be easily accessible and easy to understand. Limiting the number of options was thought to make things easier. Other studies than the present one were also presented on *minforskning.se*, which created confusion. It was perceived as demanding to find the information, which was seen as a hurdle:

It took me a very, very long time to find the right way in it… No, I got to click around and try a little here and there and try to figure it out myself, which I did at the end, but it felt a little unnecessarily annoying. (Participant 15)

##### Navigation in digital tools

Difficulties regarding navigation in the digital tools were described. Participants expressed that they were getting lost and viewed lack of an overview. These factors often led to giving up and abandonment of these tools. It was not always clear that the platforms were separate. Participants suggested that an e-mail with a link, menus, and a few clicks might make the navigation easier. Many requested an app which was thought to simplify:

If you need information, then you should get to the information within two or max three clicks. (Participant 2)

##### What to do and how to do it

Easily understandable and clear instructions were appreciated. This was highly demanded when using digital tools. Links via e-mail and simple questionnaires were also preferred rather than platforms. The lack of information was a major obstacle when using the digital tools and participants tended to give up when there were unclear expectations. However, short instructions and informational texts were preferred, while longer versions for those who were interested were suggested for improvement. Meanwhile, the instructions for the self-administered home blood test were easily understood by participants and made it easy to execute the task without further questions:

And the clearness, especially for us that are not so skilled with computers, is that…It may not be hindered because you are not that skilled with computers. It has to be an educational, clear method. “Do like this”. (Participant 4)

It was simple. It was very simple…It was just to prick the finger and put on blood droplets there, then it was…Then it was done….It can not be much simpler. (Participant 16)

##### A receipt for confirmation

There was a request for confirmation when using digital tools. Being assured that the task was completed was key. Lack of this created uncertainty and a sense of frustration:

I know that I completed some questionnaires. One in the beginning and then something at the end. But if I did it correctly or not, that I actually don’t know. I thought it felt a little unsure. (Participant 3)

##### Good computer skills enough?

The self-perceived computer skills among the participants varied but were not always described to be the most crucial factor when tackling digital tools. Many found that even experience in using digital tools was not always enough. The digital tools were still often viewed as difficult to navigate. Many found that the tools had not been previously evaluated for user friendliness. While some thought it was not developed for the “average person,” some thought it was not enough to develop it for those with such skills either:

It should appear on the first site…or be visible where you should go and then you should click through without having to…what should I say, search and wonder “what does that mean and how are they thinking here?”// It feels like it is those that do a lot of these things, that think things are obvious. Computer nerds and other things, that (laugh) take things for granted that you should understand. // Yes, you have to try it on people. //And then they have to think about all the stupid thoughts, or those that do not comprehend anything, that the must…It should be idiotproof. (Participant 17)

It was not a lot of issues with it. I am pretty digital, or I can say I am very digital, I work with a lot of digital tools in my job. So I do not think I experienced anything particular. (Participant 20)

### Synthesis

This convergent mixed methods study in preparation of the decentralized DIATEST-HELP trial illustrates the importance of the user's own motivation and needs and the safety, security, usability, and efficacy of the used technical platforms, as well as the information and support given to participants.

Our results suggest that these components are crucial to users of the tested digital tools and self-administered home blood tests. While consenting on *minforskning.se* and self-administered home blood tests were described as user-friendly in the MAUQ, as well as in the interviews, the digital tools were not perceived as either well organized or easy to navigate, which resulted in difficulties finding the information. Furthermore, the digital tools were not seen as helpful in health management, nor did they meet the participants’ expectations.

## Discussion

The results of this pilot study indicate that the willingness to use digital tools and self-administered home blood tests for study participation from home is closely related to the perceived benefits they provide to the user. This is in alignment with previous results showing that modification of digital tools to the intended users’ wants and needs increases their user engagement.^19–21^ Users of digital tools have a tendency to be willing to risk the integrity of their personal data if the tool provides benefits.^20^ Despite this, several studies in addition to our study have indicated that concerns related to integrity and misuse of data may lead to an unwillingness to use digital tools for healthcare purposes as well as for clinical trial participation.^22–25^

Our findings underscore the need for clear information and support when using digital tools. Prior studies have noted that patients may perceive electronic communications with healthcare providers as inappropriate.^26^ Consistent with our results, participants recognized the digital divide, i.e., disparities in access to digital technologies, which poses challenges for certain groups, such as older people, potentially leading to recruitment bias.^24,27^ Additional bias may stem from low digital literacy and lack of confidence in using such tools.^24,28^ To facilitate participation, we used *BankID*, a widely adopted digital identification method in Sweden, accessible to 92% of the population and 98.7% of citizens aged 18–75.^14^ Notably, even digital experienced users encountered difficulties with the tools in this study.

Our study confirms that prompt responses to inquiries are valued, aligning with previous findings in digital healthcare communication.^19^ While participants were highly motivated by access to laboratory results, such access may also induce anxiety due to uncertainty about the results’ significance.^29^ Self-administered home blood test can accelerate data collection in clinical trials.^5^ Participants preferred that healthcare professionals monitor clinical data and intervene when necessary, which is found previously.^30^

Participants emphasized the need for comprehendible information, consistent with prior research highlighting the importance of testing digital tools to ensure user understanding.^7,21,31^ Similarly, previous studies report higher user satisfaction with tools that are intuitive, clearly instructed, and user-friendly.^30,32,33^

## Strengths and limitations

A strength is the novel research tools and the study methodology, a convergent mixed methods study to evaluate these. A design using both a quantitative and qualitative approach render a broad and useful insight in further amendments and adaptations needed in preparation for a randomized controlled trial. Conducting telephone interviews may have an advantage as it has been suggested that participants are more likely to answer honestly.^34^ However, some aspects of a physical meeting, such as the dynamics in a face-to-face conversation and the possibility of interpretation of facial expressions as well as body language, are lost. One researcher (LH) conducted all the interviews. One pilot interview was conducted and transcribed verbatim. The transcript was assessed by the supervisor (LK) and LH was given constructive criticism regarding the interview both with respect to the content of the interview guide. i.e. the way the questions were put in the guide, and with respect to the interview techniques, i.e. the way the questions were asked by the researcher and how in-depth and clarifying questions were asked. The quality of the pilot interview was considered high, and no subsequent changes regarding the content of the interview guide were made after the assessment of the pilot interview. The preliminary categories were coded by two of the authors (LH and LK). When the preliminary analysis was completed, an additional researcher (KR) was added, strengthening the results further through investigator triangulation and ensuring credibility.^16^ As part of the reflexivity process, the categories were confirmed by complementing and contesting each other's readings and pre-understandings.^16^ Validity was built into the analysis by testing the preliminary categories until agreement was reached, which contributed to trustworthiness.^16^ Consolidated criteria for reporting qualitative research (COREQ)^35^ was used, which further strengthened the reliability of the study.

Limitations of the study are the incomplete questionnaire data from the MAUQ, where less than half answered this questionnaire at the end of the study, and the baseline questionnaire, especially the question on education. Furthermore, the MAUQ was not used in full as one item on the MAUQ (i.e. Q14: “The app improved my access to health care services”) was excluded, because it was not relevant to the tools evaluated in our study. The choice to use an extensive questionnaire, with a known high respondent burden to test responders’ reaction to reminders, may have reduced participant's interest in providing answers which in turn may have affected the response rate and perhaps also the participants’ general perception of the digital tools. Furthermore, generalizability outside of the Scandinavian countries is limited.

For the qualitative part of the study, generalizability is not possible. However, we believe that the transferability of our findings to a broad variety of people on their perspectives on smartphone usage, self-administered home blood tests, and digital tools is possible.

The results from this pilot study have rendered new features and adaptations that were implemented to meet user expectations of *minforskning.se*. A new version of *minforskning.se* has been launched, where instructions have been updated to be more informative and easier to read and informative videos have been added to improve usability.

## Conclusion

In conclusion, self-administered home blood tests and digital informed consent were perceived as user-friendly, but the digital tools were difficult to navigate and did not meet the participants’ expectations in their present form. This study highlights the importance of evaluating decentralized trial methodology by the intended users and thereby enabling the development of tools that are meaningful to its users, which, consequently, can facilitate trial participation and data collection. The results of this study will inform further platform development work in preparation of the decentralized DIATEST-HELP trial and may be helpful to improve the digital tools in the development of other, similar technologies for usage in clinical trials.

## Supplemental Material

sj-doc-1-dhj-10.1177_20552076251365063 - Supplemental material for Digital tools and self-administered home blood tests: A convergent mixed methods pilot study Supplemental material, sj-doc-1-dhj-10.1177_20552076251365063 for Digital tools and self-administered home blood tests: A convergent mixed methods pilot study by Lana Hebib, Emelie Gustafson Hedov, Maria Storgärds, Peder af Geijerstam, Marie Löf, Jason HY Wu, Lisa Kastbom and Karin Rådholm in DIGITAL HEALTH
