# Supplementary material for: Digital tools and self-administered home blood tests: A convergent mixed methods pilot study
Source: Digit Health. 2025 Jul 31;11:20552076251365063. doi: 10.1177/20552076251365063 (PMC12317230; doi:10.1177/20552076251365063)
Supplement: sj-doc-1-dhj-10.1177_20552076251365063 - Supplemental material for Digital tools and self-administered home blood tests: A convergent mixed methods pilot study [file sj-doc-1-dhj-10.1177_20552076251365063.doc]

**SUPPLEMENTARY MATERIAL**

**mHealth App Usability Questionnaire (MAUQ)**

**for Standalone mHealth Apps Used by Patients**

| # | Statements | N/A | 1 2 3 4 5 6 7 |
| --- | --- | --- | --- |
| 1. | The app was easy to use. | ☐ | DISAGREE ☐ ☐ ☐ ☐ ☐ ☐ ☐ AGREE |
| 2. | It was easy for me to learn to use the app. | ☐ | DISAGREE ☐ ☐ ☐ ☐ ☐ ☐ ☐ AGREE |
| 3. | The navigation was consistent when moving between screens. | ☐ | DISAGREE ☐ ☐ ☐ ☐ ☐ ☐ ☐ AGREE |
| 4. | The interface of the app allowed me to use all the functions (such as entering information, responding to reminders, viewing information) offered by the app. | ☐ | DISAGREE ☐ ☐ ☐ ☐ ☐ ☐ ☐ AGREE |
| 5. | Whenever I made a mistake using the app, I could recover easily and quickly. | ☐ | DISAGREE ☐ ☐ ☐ ☐ ☐ ☐ ☐ AGREE |
| 6. | I like the interface of the app. | ☐ | DISAGREE ☐ ☐ ☐ ☐ ☐ ☐ ☐ AGREE |
| 7. | The information in the app was well organized, so I could easily find the information I needed. | ☐ | DISAGREE ☐ ☐ ☐ ☐ ☐ ☐ ☐ AGREE |
| 8. | The app adequately acknowledged and provided information to let me know the progress of my action. | ☐ | DISAGREE ☐ ☐ ☐ ☐ ☐ ☐ ☐ AGREE |
| 9. | I feel comfortable using this app in social situations. | ☐ | DISAGREE ☐ ☐ ☐ ☐ ☐ ☐ ☐ AGREE |
| 10. | The amount of time involved in using this app has been fitting for me. | ☐ | DISAGREE ☐ ☐ ☐ ☐ ☐ ☐ ☐ AGREE |
| 11. | I would use this app again. | ☐ | DISAGREE ☐ ☐ ☐ ☐ ☐ ☐ ☐ AGREE |
| 12. | Overall, I am satisfied with this app. | ☐ | DISAGREE ☐ ☐ ☐ ☐ ☐ ☐ ☐ AGREE |
| 13. | The app would be useful for my health and well-being. | ☐ | DISAGREE ☐ ☐ ☐ ☐ ☐ ☐ ☐ AGREE |
| 14. | The app improved my access to healthcare services. | ☐ | DISAGREE ☐ ☐ ☐ ☐ ☐ ☐ ☐ AGREE |
| 15. | The app helped me manage my health effectively. | ☐ | DISAGREE ☐ ☐ ☐ ☐ ☐ ☐ ☐ AGREE |
| 16. | This app has all the functions and capabilities I expected it to have. | ☐ | DISAGREE ☐ ☐ ☐ ☐ ☐ ☐ ☐ AGREE |
| 17. | I could use the app even when the Internet connection was poor or not available. | ☐ | DISAGREE ☐ ☐ ☐ ☐ ☐ ☐ ☐ AGREE |
| 18. | This mHealth app provides an acceptable way to receive healthcare services, such as accessing educational materials, tracking my own activities, and performing self-assessment. | ☐ | DISAGREE ☐ ☐ ☐ ☐ ☐ ☐ ☐ AGREE |

In this questionnaire, 1 - strongly disagree, 2 – disagree, 3 – somewhat disagree, 4 – neither agree nor disagree, 5 – somewhat agree, 6 – agree, 7 – strongly agree

To determine the usability of an app, calculate the total and determine the average of the responses to all statements. The higher the overall average, the higher the usability of the app.

Citera: Zhou L, Bao J, Setiawan A, Saptono A, Parmanto B, (2019), "The mHealth App Usability Questionnaire (MAUQ): Development and Validation Study", *JMIR mHealth and uHealth*, 7(4):e11500. DOI: 10.2196/11500. PMID: 30973342

**Additional study specific questions added to mHealth App Usability Questionnaire (MAUQ)**

| A1. | I feel comfortable consenting to participate in research studies digitally via minforskning.se. | ☐ | DISAGREE ☐ ☐ ☐ ☐ ☐ ☐ ☐ AGREE |
| --- | --- | --- | --- |
| A2. | It was easy to join the study and get reminders via minforskning.se. | ☐ | DISAGREE ☐ ☐ ☐ ☐ ☐ ☐ ☐ AGREE |
| B1. | I feel comfortable to prick myself in a finger at home (for the blood sample). | ☐ | DISAGREE ☐ ☐ ☐ ☐ ☐ ☐ ☐ AGREE |
| B2 | The instructions for the home blood tests were clear and easy to understand. |  | DISAGREE ☐ ☐ ☐ ☐ ☐ ☐ ☐ AGREE |
| B3. | It went well getting enough blood to fill the circles for the home blood tests. | ☐ | DISAGREE ☐ ☐ ☐ ☐ ☐ ☐ ☐ AGREE |

**Leave comments about the digital health tool:**

# Interview guide

## Background

- Oral consent (voluntary to participate, you may withdraw your consent at any time, no personal information is recorded and nothing of what you say can be linked to you but will be presented on a group level)
- Gender
- Age
- Living situation (Urban area? Countryside?)
- Social situation (Living alone? Cohabitation? Partner? Children?)
- Occupation/Educational level

## Use of cellphone in general

How often do you use the cellphone?

- How much do you use your cellphone?
- What do you use your cellphone for?
- How many notifications do you usually get each day?
- Can you reflect about what makes you react, and in what way you react, to different kinds of notifications? Which kinds of notifications do you react to? How do you react to these notifications?
- Do you have any experience of the use of mobile apps as a support in chronic diseases? If yes, how? What is your attitude towards the use of mobile applications as a support in your daily life/to registrate the way you are feeling? I would like you to reflect about what you find would make the optimal care/healthcare/support in chronic disease!

## About the experience of self-administrated home blood tests

- What were your experiences of home blood testing?
- What was positive about home blood testing?
- What was negative about home blood testing?
- If you could give suggestions to improvements regarding home blood testing, what would they be?
- Which possibilities do you see with home blood testing in research projects?
- Which difficulties do you see with home blood testing in research projects?

## About the digital tools

- What was your experience of the use of the platform *minforskning.se*?
- What worked well? What worked less well?
- What was your experience of the use of the phone application S*ymptoms*?
- What worked well? What worked less well?
- How would the digital tools (*minforskning.se, Symptoms*) be able to develop to work even better?
- Which factors do you find simplify the use of a digital tool?
- Which difficulties do you see with the use of a digital tool?

Is there anything that you would like to add regarding home blood testing or about digital tools?

**Supplementary figures**


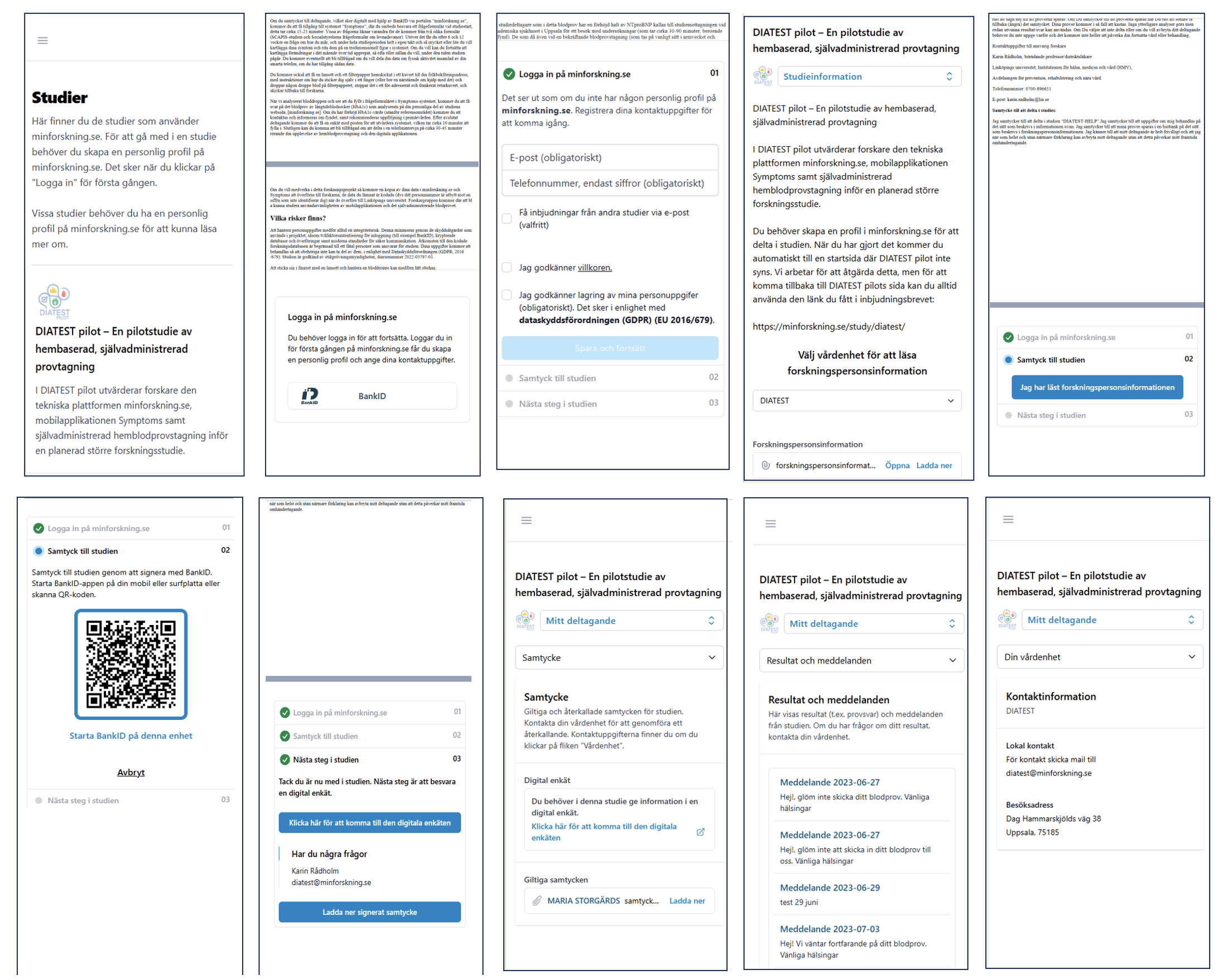


**Figure 1. Screenshots from the *Minforskning.se* platform showing enrollment process in the DIATEST pilot study**

**Top row (first row):** These images show the process of joining the DIATEST pilot study via the Swedish research platform *Minforskning.se*:
**Left panel:** Overview of available studies. The description for DIATEST pilot reads:
"DIATEST pilot – A pilot study of home-based, self-administered sampling. In DIATEST pilot, researchers evaluate the technical platform *Minforskning.se*, the mobile application Symptoms, and self-administered home blood sampling ahead of a planned larger research study."
**Middle panel:** Informed consent is provided digitally by signing with the Swedish electronic identification system BankID.
**Right panel:** Confirmation that the participant is enrolled in the study. The next step is to complete a digital questionnaire. Contact details for the study coordinator and a button to download the signed consent form are also displayed.

**Bottom row (second row):**
**Left panel:** Participants scan a QR code to log in using *BankID* on a different device.
**Middle panel:** Consent is confirmed, and participants are thanked for joining the study. A button provides access to the digital questionnaire.
**Right panel:** Overview of the participant’s messages, study results, and contact information.


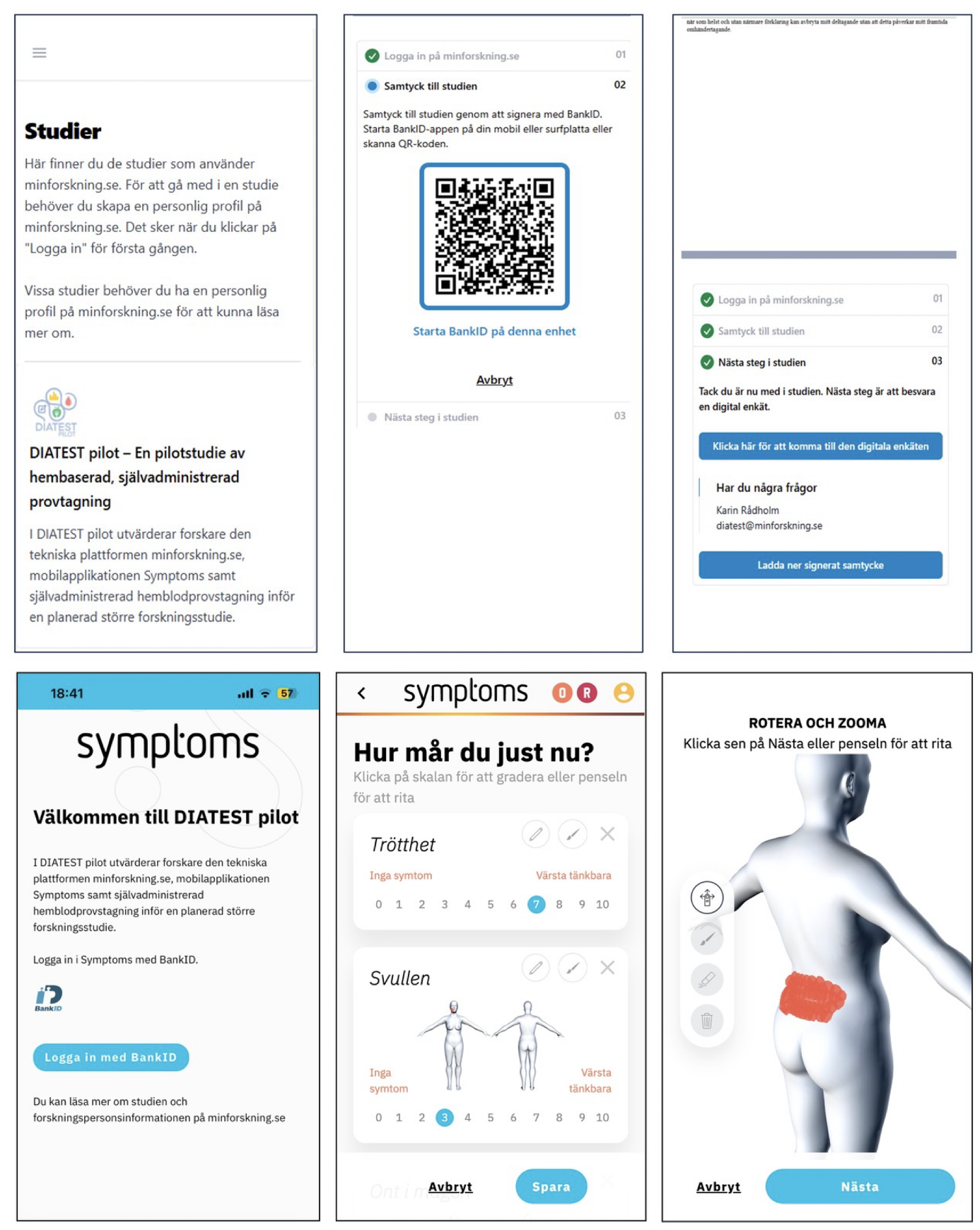


**Figure 2. Screenshots from the *Symptoms* mobile application used in the DIATEST pilot study**

These images show the user interface of the Symptoms app, which is used in the DIATEST pilot study for digital symptom reporting:

Top row (left to right):

• **Welcome screen.** Text translation:

"Welcome to DIATEST pilot. In DIATEST pilot, researchers evaluate the technical platform *Minforskning.se*, the mobile application *Symptoms*, and self-administered home blood sampling ahead of a planned larger research study."

• **Symptom severity rating interface.** Users rate current symptoms from 0 (no symptoms) to 10 (worst imaginable). Examples shown: Trötthet (Fatigue) and Svullen (Swelling), with anatomical location selection.

• **3D symptom localization tool.** A rotatable model where users draw symptom areas using a digital brush.

**Bottom panel:**

• Free-text reporting interface. Users can describe additional symptoms in their own words. The interface includes a keyboard input field and controls to cancel or proceed.

**Supplementary methods**

Copyright

Licences for usage of minforskning.se and Symptoms were obtained. Permission for using the MAUQ was obtained, and the questionnaire has been professionally translated, and reported back to the MAUQ team.
